# Editorial: Dispersal ecology of land plants: striving towards a more universal understanding

**DOI:** 10.3389/fpls.2025.1650901

**Published:** 2025-07-22

**Authors:** M. Teresa Boquete, Irene Bisang, F. Javier Valverde

**Affiliations:** ^1^ Unidad de Botánica, Departamento de Biología, Facultad de Ciencias, Universidad Autónoma de Madrid, Madrid, Spain; ^2^ Centro de Investigación en Biodiversidad y Cambio Global (CIBC), Universidad Autónoma de Madrid, Madrid, Spain; ^3^ Department of Botany, Swedish Museum of Natural History, Stockholm, Sweden; ^4^ Área de Botánica, Departamento de Biología Vegetal y Ecología, Universidad de Sevilla, Sevilla, Spain

**Keywords:** embryophytes, plant dispersal, community composition, population structure, distribution ranges, comparative analyses, community structure

Plant dispersal is a fundamental ecological process involving the movement of individuals and genes across spatial and temporal scales at different life stages. Although this process operates at the individual level, it has far-reaching consequences at higher organizational levels through its impact on the structure and dynamics of entire populations and communities (Cousens et al., 2008; Snell et al., 2019; Rogers et al., 2021; Allbee et al., 2023). Plant dispersal capacities also govern plant distributional ranges and abundance over space and time which contributes to shaping biodiversity at an even broader scale (Lönnell et al., 2014; Barbé et al., 2016; Alzate and Onstein, 2022; Petrocelli et al., 2024). Despite its ecological and evolutionary significance, it is striking that research on plant dispersal has largely focused on spermatophytes (seed plants). Consequently, our understanding of dispersal mechanisms and their consequences remains limited for other major terrestrial plant groups, such as bryophytes and pteridophytes. These spore-producing plants play essential roles in many ecosystems (Gibby, 2023) and their spatial distribution and range are shaped by distinct dispersal drivers and patterns compared to seed plants (Kessler, 2010; Lönnell et al., 2014; Barbé et al., 2016; Almeida et al., 2021). This gap in knowledge hinders our ability to develop a more comprehensive understanding of how plant dispersal shapes populations, communities, and, more broadly, global biodiversity patterns.

This Research Topic brings together five original research articles that broaden our perspective on plant dispersal: two focused on bryophytes (Lara et al.; Bisang et al.), two on flowering plants (Moss and Evans; Zheng et al.), and one comparative study bridging both groups (Fichant et al.).

Fichant et al. conducted what appears to be the first comparative meta-analysis of genetic spatial patterns driven by the dispersal of male gametes (sperm cells and pollen) and diaspores (spores and seeds) in both bryophytes and spermatophytes. Focusing on European populations of 11 bryophyte and 17 spermatophyte species genotyped using both nuclear (nDNA) and chloroplast DNA (cpDNA), they found significantly lower genetic differentiation between northern and southern populations in bryophytes when analyzing cpDNA, but not with nDNA. This indicates that spores disperse more effectively than seeds across major geographic barriers such as the Alps and the Pyrenees, leading to a more homogeneous genetic structure. These differences in dispersal ability have influenced post-glacial migration and current distribution patterns. These findings are critical for predicting how different plant groups may shift their ranges in response to ongoing climate change.

The results by Fichant et al. support the notion that bryophytes are strong long-distance dispersers. This has led to the assumption that bryophytes often show very broad geographic ranges or even intercontinental disjunctions. However, evidence accumulates that many presumed widespread species actually comprise species complexes. Lara et al. advanced this line of inquiry by combining molecular and morphological data to uncover pseudo-cryptic speciation in the moss *Lewinskya firma* (Venturi) F. Lara, Garilleti & Goffinet. Previously believed to span East Africa and South India, this species was found to consist of two distinct lineages: *L. firma*, restricted to East Africa, and *L. afroindica*, distributed across South and East Africa and South India. Phylogenetic analyses support a recent, single long-distance dispersal event from East or South Africa to India. This work underscores the importance of long-distance dispersal in shaping plant distributions, while also emphasizing the need of detailed taxonomic and phylogenetic work to accurately interpret species biogeographic histories.

Another study in this Research Topic beyond Fichant et al. includes pre-zygoytic aspects of dispersal, recognizing that plant dispersal is closely tied to reproduction (Bisang et al.). Sexual reproduction in bryophytes is often hampered by the lack of sex expression (i.e., production of reproductive organs), and the presence and proximity of male and female individuals. They investigated how climatic and topographic variables may influence these reproductive traits and thus affect bryophyte dispersal. While the geographic distribution of sexes and levels of sex expression were not significantly shaped by large-scale environmental factors, actual sexual reproduction was responsive to these variables. This suggests that constraints such as sex-ratio imbalance and spatial segregation may limit local reproduction but have less impact on long-distance dispersal. In contrast to seed plants, bryophytes may require only occasional sexual events to enable effective dispersal and maintain genetic connectivity across large areas.

Turning to seed plants, Zheng et al. compared the impact of dispersal and post-dispersal environmental filtering on plant community assemblages along an elevational gradient in a dryland mountain range. Comparing woody and herbaceous communities, they found that environmental filtering and dispersal limitation together explained 33–77% of species turnover across the 1,420 m gradient. In woody plants, environmental variables had a stronger effect than dispersal variables. Jointly, the two filters explained markedly more of the species turnover in woody than herbaceous plants. These results highlight that even within a single plant lineage, species with different life-history strategies are shaped by distinct processes, underscoring the complexity of community assembly and biodiversity patterns along environmental gradients.

Finally, Moss and Evans addressed the effect of climate change on the potential of male gamete dispersal and plant reproduction in several species of agronomical interest. The authors experimentally demonstrate that even modest increases in temperature (e.g., 1.5°C) can significantly alter floral abundance, seed production, and the dynamics of plant–pollinator relationships, while the responses differed between species. These changes raise important concerns about how altered reproductive outputs under climate change may feedback into dispersal processes and community structure, especially in systems reliant on successful pollination for seed production and population persistence.

To gain more widely applicable life history theories for plants, it is critical to go beyond the plant groups that currently underpin generalizations in the field. By integrating perspectives on male gamete and diaspore dispersal in both spore- and seed-producing plants, the studies in this Research Topic underscore the diversity of dispersal strategies and the ecological and evolutionary consequences they entail. The findings reveal that dispersal is not a uniform process but one deeply influenced by reproductive biology, life-history traits, and environmental context. Importantly, these studies also emphasize the value of combining field and experimental studies with molecular and modelling approaches to better understand the mechanisms driving plant distribution and diversity. As climate change continues to reshape ecosystems globally, advancing our understanding of plant dispersal across taxonomic groups will be essential for predicting future biodiversity patterns and informing conservation strategies.

## References

[B1] Allbee S. A. Rogers H. S. Sullivan L. L. (2023). The effects of dispersal, herbivory, and competition on plant community assembly. Ecology 104, e3859. doi: 10.1002/ecy.3859, PMID: 36054771 PMC10078099

[B2] Almeida T. E. Salino A. Dubuisson J.-Y. Hennequin S. (2021). Insights into long-distance dispersal and ecological and morphological evolution in the fern genus Microgramma from phylogenetic inference. Bot. J. Linn. Soc. 196, 294–312. doi: 10.1093/botlinnean/boaa107

[B3] Alzate A. Onstein R. E. (2022). Understanding the relationship between dispersal and range size. Ecol. Lett. 25, 2303–2323. doi: 10.1111/ele.14089, PMID: 36001639

[B4] Barbé M. Fenton N. J. Bergeron Y. (2016). So close and yet so far away: long-distance dispersal events govern bryophyte metacommunity reassembly. J. Ecol. 104, 1707–1719. doi: 10.1111/1365-2745.12637

[B5] Cousens R. Dytham C. Law R. (2008). “Propagule dispersal and the spatial dynamics of populations and communities,” in Dispersal in Plants: A Population Perspective. Eds. Cousens R. Dytham C. Law R. (Oxford: Oxford University Press). doi: 10.1093/acprof:oso/9780199299126.003.0007

[B6] Gibby M. (2023). “Bryophytes and pteridophytes: spore-bearing land plants,” in The Living Planet: The State of the World’s Wildlife. Ed. Maclean N. (Cambridge University Press, Cambridge), 37–64. doi: 10.1017/9781108758826.004

[B7] Kessler M. (2010). “Biogeography of ferns,” in Fern Ecology. Eds. Sharpe J. M. Mehltreter K. Walker L. R. (Cambridge University Press, Cambridge), 22–60. doi: 10.1017/CBO9780511844898.003

[B8] Lönnell N. Jonsson B. G. Hylander K. (2014). Production of diaspores at the landscape level regulates local colonization: an experiment with a spore-dispersed moss. Ecography 37, 591–598. doi: 10.1111/j.1600-0587.2013.00530.x

[B9] Petrocelli I. Alzate A. Zizka A. Onstein R. E. (2024). Dispersal-related plant traits are associated with range size in the Atlantic Forest. Diversity Distributions 30, e13856. doi: 10.1111/ddi.13856

[B10] Rogers H. S. Donoso I. Traveset A. Fricke E. C. (2021). Cascading impacts of seed disperser loss on plant communities and ecosystems. Annu. Rev. Ecol. Evol. Syst. 52, 641–666. doi: 10.1146/annurev-ecolsys-012221-111742

[B11] Snell R. S. Beckman N. G. Fricke E. Loiselle B. A. Carvalho C. S. Jones L. R.. (2019). Consequences of intraspecific variation in seed dispersal for plant demography, communities, evolution and global change. AoB Plants 11, plz016. doi: 10.1093/aobpla/plz016, PMID: 31346404 PMC6644487

